# Lectotype designations and nomenclatural changes in *Xylographus* Mellié (Coleoptera, Ciidae)

**DOI:** 10.3897/zookeys.374.6553

**Published:** 2014-01-28

**Authors:** Vivian Eliana Sandoval-Gómez, Cristiano Lopes-Andrade, John F. Lawrence

**Affiliations:** 1 Programa de Pós-Graduação em Entomologia, Departamento de Entomologia, Universidade Federal de Viçosa, 36570-900, Viçosa, Minas Gerais, Brazil; 2 Departamento de Biologia Animal, Universidade Federal de Viçosa, 36570-900, Viçosa, Minas Gerais, Brazil; 3 Australian National Insect Collection, CSIRO Ecosystem Sciences, GPO Box 1700, Canberra, ACT 2601, Australia

**Keywords:** Ciid, minute tree-fungus beetle, Orophiini, type material

## Abstract

We designate lectotypes and propose nomenclatural changes in *Xylographus* Mellié (Coleoptera, Ciidae) based on type specimens deposited in the Museum of Comparative Zoology (USA), Museum für Naturkunde Berlin (Germany), the Natural History Museum (UK), Muséum d’Histoire Naturelle de la Ville de Genève (Switzerland), Muséum National d’Histoire Naturelle (France), Naturhistoriska Riksmuseet (Sweden) and Naturhistorisches Museum Wien (Austria). We designate lectotypes for the following species: *Cis fultoni* Broun, 1886, *Xylographus anthracinus* Mellié, 1849, *X. bicolor* Pic, 1916, *X. brasiliensis* Pic, 1916, *X. ceylonicus* Ancey, 1876, *X. contractus* Mellié, 1849, *X. corpulentus* Mellié, 1849, *X. dentatus* Pic, 1922, *X. gibbus* Mellié, 1849, *X. hypocritus* Mellié, 1849, *X. javanus* Pic, 1937, *X. lemoulti* Pic, 1916, *X. longicollis* Pic, 1922, *X. madagascariensis* Mellié, 1849, *X. nitidissimus* Pic, 1916, *X. perforatus* Gerstaecker, 1871, *X. porcus* Gorham, 1886, *X. punctatus* Mellié, 1849, *X. ritsemai* Pic, 1921, *X. rufescens* Pic, 1921, *X. rufipennis* Pic, 1934, *X. rufipes* Pic, 1930, *X. seychellensis* Scott, 1926, *X. subopacus* Pic, 1929, *X. subsinuatus* Pic, 1916, *X. suillus* Gorham, 1886, *X. testaceitarsis* Pic, 1916 and *X. tomicoides* Reitter, 1902. We propose the following **syn. n.** (senior synonym listed first): *X. anthracinus* = *X. testaceitarsis*, *X. brasiliensis* = *X. lucasi* Lopes-Andrade & Zacaro, *X. corpulentus* = *X. lemoulti* and *X. richardi* Mellié, *X. madagascariensis* = *X. eichelbaumi* Reitter, *X. rufipennis*, *X. seychellensis* Scott and *X. tarsalis* Fåhraeus, *X. nitidissimus* = *X. longicollis*, *X. subsinuatus* = *X. rufescens*. We exclude three species from *Xylographus*: *Cis renominatus*, **nom. n.** (for *X. dentatus* Pic, 1922, not *C. dentatus* Mellié, 1849), *Paratrichapus fultoni* (Broun, 1886), **comb. n.** and *P. javanus* (Pic, 1937), **comb. n.**

## Introduction

*Xylographus* Mellié (Coleoptera, Ciidae, Orophiini) is a genus of minute tree-fungus beetles with 36 described species, occurring in most continental and insular lands of tropical and subtropical regions (Lawrence and Lopes-Andrade 2010, Sandoval-Gómez et al. 2011). The name *Xylographus* was mentioned for the first time in the catalogue of Dejean (1835), but became available only after its description by Mellié (1847). Six species names were cited in the original description of the genus, but only one of them was available, *Cis bostrichoides* Dufour, 1843, being its type species by monotypy. Afterwards, Mellié (1849) described the other five species and proposed three more, respectively: *Xylographus anthracinus* Mellié, 1849, *Xylographus contractus* Mellié, 1849, *Xylographus corpulentus* Mellié, 1849, *Xylographus gibbus* Mellié, 1849, *Xylographus hypocritus* Mellié, 1849, *Xylographus madagascariensis* Mellié, 1849, *Xylographus punctatus* Mellié, 1849 and *Xylographus richardi* Mellié, 1849. Moreover, he synonymized *Cis cribatus* Lucas, 1849 with *Xylographus bostrichoides*.

In the late XIX century, six species of *Xylographus* were described: *Xylographus perforatus* Gerstaecker, 1871, *Xylographus tarsalis* Fåhraeus, 1871, *Xylographus ceylonicus* Ancey, 1876, *Xylographus latirostris* Gorham, 1886, *Xylographus porcus* Gorham, 1886 and *Xylographus suillus* Gorham, 1886. *Xylographus latirostris* was later transferred to *Ceracis* Mellié, 1847 by Lawrence (1971) and *Cis fultoni* Broun, 1886 to *Xylographus* by Kuschel (1990).

The first half of the XX century was marked by a considerable increase in number of *Xylographus*, with the description of 19 species. Edmund Reitter described three species: *Xylographus tomicoides* Reitter, 1902, *Xylographus eichelbaumi* Reitter, 1908 and *Xylographus globipennis* Reitter, 1911. Maurice Pic was the most prolific author, describing 14 species: *Xylographus bicolor* Pic, 1916a, *Xylographus brasiliensis* Pic, 1916a, *Xylographus lemoulti* Pic, 1916b, *Xylographus nitidissimus* Pic, 1916a, *Xylographus subsinuatus* Pic, 1916b, *Xylographus testaceitarsis* Pic, 1916a, *Xylographus ritsemai* Pic, 1921, *Xylographus rufescens* Pic, 1921, *Xylographus dentatus* Pic, 1922, *Xylographus longicollis* Pic, 1922, *Xylographus subopacus* Pic, 1929, *Xylographus rufipes* Pic, 1930, *Xylographus rufipennis* Pic, 1934 and *Xylographus javanus* Pic, 1937. However, these species are difficult to recognize, because their original descriptions are very brief, lacking adequate diagnostic characteristics and some of them may constitute synonyms of species previously proposed by other authors (Sandoval-Gómez et al. 2011). Scott (1926) described *Xylographus seychellensis*, but indicated that it could be a synonym of one of the Afrotropical species described by Pic, which he could not examine. Blair (1940) described *Xylographus bynoei*.

In the second half of the XX century only two species were described: *Xylographus nakanei* Nobuchi, 1955 and *Xylographus scheerpeltzi* Nobuchi & Wada, 1956. *Xylographus nakanei* was proposed as junior synonym of *Paraxestocis unicornis* Miyatake, 1954 by Kawanabe (1995). Finally, after almost a half century without new descriptions of *Xylographus*, *Xylographus lucasi* was described by Lopes-Andrade and Zacaro (2003). Ferrer (1997) designated lectotypes of *Xylographus* species described by Fåhraeus (1871) and Reitter (1908). Later, in a paper on the Afrotropical *Xylographus globipennis*, its lectotype was designated (Sandoval-Gómez et al. 2011).

Recently we had the opportunity to examine type material of the most important historical collections of *Xylographus*. During this work, we noted that some species should be excluded from the genus and several synonyms were recognized. It is necessary to propose these nomenclatural acts now, before finishing the revision of *Xylographus*, because some names will soon be cited in ecological, cytotaxonomic and phylogenetic works on ciids. As most descriptions of *Xylographus* are based on syntypes, lectotype designations are necessary to fix clearly the concept of the names and to ensure the universal and consistent interpretation of them.

## Material and methods

We examined 195 type specimens of *Xylographus* from the following institutions (preceded by acronyms used in this paper):

MCZ Museum of Comparative Zoology, Harvard University (Cambridge, Massachusetts, United States)

MFNB Museum für Naturkunde Berlin (Berlin, Germany)

MHNG Muséum d’histoire naturelle de la ville de Genève (Geneva, Switzerland)

MNHN Muséum National d’Histoire Naturelle (Paris, France)

NHM The Natural History Museum (London, United Kingdom)

NHMW Naturhistorisches Museum Wien (Wien, Austria)

NHRS Naturhistoriska Riksmuseet (Stockholm, Sweden)

We used the generic features of *Xylographus* cited by Sandoval-Gómez et al. (2011), the most important features proposed by Lawrence (1971) to recognize *Cis*, and the original description of *Paratrichapus* by Scott (1926), for making decisions on generic placement. *Paratrichapus* was described as having a 3-3-3 tarsal formula, but after studying its type material and images of microscope slide preparations by Hugh Scott, we observed that it was certainly 4-4-4 as in all other ciids. *Xylographus* and *Paratrichapus* are morphologically similar, so we propose the characteristics stated on Table 1 to differentiate them.

**Table 1.** Main differences between *Xylographus* Mellié and *Paratrichapus* Scott.

Features	*Xylographus*	*Paratrichapus*
left mandible usually bearing an upward tooth in males	present in most species	absent
first labial palpomere	elongate, as long as or longer than the second one	shorter than the second one
pronotum punctation	dual, fine to coarse	single, always deep and coarse
prosternum	concave	biconcave
elytral length/elytral width	less than 1.15	more than 1.15
elytral length/pronotal length	less than 1.4	more than 1.4
protibial socketed spines	extending from the apex to almost its base	extending from the apex to at most its middle
first and second tarsomeres	subconical and well separated	subcylindrical and contiguous

We have not located the types of *Xylographus bostrichoides* and *Xylographus richardi*. And we did not have access to type material of *Xylographus scheerpeltzi*. In the case of *Xylographus bostrichoides*, we had at hand several named historical specimens, including those used for its redescription by Mellié (1849). In the case of *Xylographus richardi*, we had only a named specimen for examination. The description of *Xylographus scheerpeltzi* is adequately detailed and includes information on the morphology of sclerites of male abdominal terminalia. In all other cases, we had access to the original type series and dissected male abdominal terminalia whenever necessary and possible. The morphology of sclerites of male abdominal terminalia of Ciidae is stable intraspecifically and distinctly varies interspecifically, even between closely related species (Antunes-Carvalho and Lopes-Andrade 2013, Oliveira et al. 2013).

We propose ten synonymies among the currently available names in *Xylographus*. For us, these are the most obvious cases that need solution. These names were proposed based on slight color differences (for instance, those observed in teneral adults), subtle variations of male secondary sexual characteristics or based only on females. A single author, Maurice Pic, was responsible for half of the names here recognized as junior synonyms. He is known for having proposed thousands of new names of beetles based mostly on anecdotal descriptions and small type-series. Lack of access to type material was also a great problem. Scott (1926) described *Xylographus seychellensis* stating that he did it with some hesitation, because he has not examined possible conspecifics, as *Xylographus madagascariensis* and *Xylographus eichelbaumi*, the senior and a junior synonym proposed here, respectively. The same was true to *Xylographus lucasi*, whose authors (Lopes-Andrade and Zacaro 2003) described it without examining the type of *Xylographus brasiliensis*, recognized here as its senior synonym.

A complete list of *Xylographus* species is given in alphabetical order. Type-locality and synonyms, if any, are given for each species. Type series and type material of its synonyms are given only for species that we could examine in museums. Syntypes of species treated in this work were almost all labeled as lectotypes and paralectotypes by John F. Lawrence in 1965, but they were not officially designated in the literature. We reexamined all specimens and preferred to maintain Lawrence’s labels in most cases to avoid future inconsistencies. We designated a lectotype in cases where a single specimen was located and the author of the species name did not state whether there was one or more than one specimen in the type series. We consider a specimen to be the holotype only when the author clearly stated there was a single specimen available for description. When exact label data are listed, a backslash (\) separates individual labels. Data in square brackets were added for clarification. Remarks are provided for some species.

## Taxonomic synopsis

*Xylographus anthracinus* Mellié, 1849

*Xylographus testaceitarsis* Pic, 1916, **syn. n.**

*Xylographus bicolor* Pic, 1916

*Xylographus bostrichoides* (Dufour, 1843)

*Cis cribatus* Lucas, 1849

*Xylographus bostrichoides* var. *aubei* Mellié, 1849

*Xylographus brasiliensis* Pic, 1916

*Xylographus lucasi* Lopes-Andrade & Zacaro, 2003, **syn. n.**

*Xylographus bynoei* Blair, 1940

*Xylographus ceylonicus* Ancey, 1876

*Xylographus contractus* Mellié, 1849

*Xylographus corpulentus* Mellié, 1849

*Xylographus lemoulti* Pic, 1916, **syn. n.**

*Xylographus richardi* Mellié, 1849, **syn. n.**

*Xylographus gibbus* Mellié, 1849

*Xylographus globipennis* Reitter, 1911

*Xylographus hypocritus* Mellié, 1849

*Xylographus madagascariensis* Mellié, 1849

*Xylographus eichelbaumi* Reitter, 1908, **syn. n.**

*Xylographus rufipennis* Pic, 1934, **syn. n.**

*Xylographus seychellensis* Scott, 1926, **syn. n.**

*Xylographus tarsalis* Fåhraeus, 1871, **syn. n.**

*Xylographus nitidissimus* Pic, 1916

*Xylographus longicollis* Pic, 1922, **syn. n.**

*Xylographus perforatus* Gerstaecker, 1871

*Xylographus porcus* Gorham, 1886

*Xylographus punctatus* Mellié, 1849

*Xylographus ritsemai* Pic, 1921

*Xylographus rufipes* Pic, 1930

*Xylographus scheerpeltzi* Nobuchi & Wada, 1956

*Xylographus subopacus* Pic, 1929

*Xylographus subsinuatus* Pic, 1916

*Xylographus rufescens* Pic, 1921, **syn. n.**

*Xylographus suillus* Gorham, 1886

*Xylographus tomicoides* Reitter, 1902

### Excluded species

*Cis renominatus*, **nom. n.**

*Xylographus dentatus* Pic, 1922, not *Cis dentatus* Mellié, 1849.

*Paratrichapus fultoni* (Broun, 1886), **comb. n.**

*Cis fultoni* Broun, 1886

*Paratrichapus javanus* (Pic, 1937), **comb. n.**

*Xylographus javanus* Pic, 1937

### Species accounts

#### Xylographus anthracinus

Mellié, 1849

http://species-id.net/wiki/Xylographus_anthracinus

Xylographus anthracinus Mellié 1849: 222, pl. 9, fig. 17. Type-locality: Madagascar.
Xylographus testaceitarsis Pic 1916: 13., **syn. n.** Type-locality: Mahatsinjo, Madagascar.

##### Type series.

MADAGASCAR: male lectotype (MNHN), here designated, labeled: “*Anthracinus* Dup. Madagascar.[handwritten] \ Ex-Musæo Mniszech [printed] \ [red label] LECTOTYPE *Xylographus anthracinus* Mellié [handwritten]”; 2 female paralectotypes (MNHN), labeled: “*anthracinus* (ex coll. Chev.) [handwritten] \ Mellié vidit [handwritten] \ [yellow label] PARALECTOTYPE *Xylographus anthracinus* Mellié [handwritten]”; 2 male paralectotypes (MHNG), labeled: “Coll. Melly [printed] \ [yellow label] PARALECTOTYPE *Xylographus anthracinus* Mellié [handwritten]”.

##### Type material of the junior synonym.

MADAGASCAR: male lectotype (MNHN) of *Xylographus testaceitarsis* Pic 1916, here designated, labeled: “MAHATSINJO près Tananarive [printed] \ Type [handwritten] \ *testaceitarsis* Pic [handwritten] \ [red label] LECTOTYPE *Xylographus testaceitarsis* Pic [handwritten]”; 1 male and 3 female paralectotypes (MNHN), labeled: “MAHATSINJO près Tananarive [printed] \ [yellow label] PARALECTOTYPE *Xylographus testaceitarsis* Pic [handwritten]”.

##### Remarks.

There is no morphological difference between the lectotype of *Xylographus anthracinus* and the lectotype of *Xylographus testaceitarsis*. They are males of about the same size and with secondary sexual characteristic similarly developed. We have also dissected and compared sclerites of their abdominal terminalia and noted no difference.

#### Xylographus bicolor

Pic, 1916

http://species-id.net/wiki/Xylographus_bicolor

Xylographus bicolor Pic 1916: 13. Type-locality: Mahatsinjo, Madagascar.

##### Type series.

MADAGASCAR: male lectotype (MNHN), here designated, labeled: “MAHATSINJO près Tananarive [printed] \ Type [handwritten] \ *bicolor* Pic [handwritten] \ [red label] LECTOTYPE *Xylographus bicolor* Pic [handwritten]”.

#### Xylographus bostrichoides

(Dufour, 1843)

http://species-id.net/wiki/Xylographus_bostrichoides

Cis bostrichoides (Dufour 1843: 93). Type-locality: Vallée d’Ossan, France.
Cis cribatus Lucas 1849: 469. Junior synonym. Type-locality:  Alger, Algeria.
Xylographus bostrichoides var. *aubei*Mellié 1849: 232. Junior synonym. Type-locality: Pyrénées, France.

##### Remarks.

Unfortunately we did not find the type material of Dufour in the MNHN. We have found only specimens used by Mellié (1849) to redescribe this species and to describe its variety *aubei*, and dozens of specimens that do fit the currently accepted species limits. Müller et al. (2001) labeled one specimen deposited in MFNB as syntype of *Xylographus bostrichoides*. However, after studying this specimen, we determined it is a member of Scolytinae (Curculionidae) and fits neither the original description by Dufour (1843) nor the redescription by Mellié (1849). Therefore, a lectotype is not designated here.

#### Xylographus brasiliensis

Pic, 1916

http://species-id.net/wiki/Xylographus_brasiliensis

Xylographus brasiliensis Pic 1916: 13. Type-locality: Rio Verde, Brazil.
Xylographus lucasi Lopes-Andrade and Zacaro 2003: 1. **syn. n.** Type-locality: Venda Nova do Imigrante, Espírito Santo, Brazil.

##### Type series.

BRASIL: female lectotype (MNHN), here designated, labeled: “Bresil. Goyaz. Rio Verde [printed] \ *Xylographus* [handwritten] \ Type [handwritten] \ *Brasiliensis* Pic [handwritten] \ [red label] LECTOTYPE *Xylographus brasiliensis* Pic [handwritten]”.

##### Type material of the junior synonym.

See Lopes-Andrade and Zacaro (2003).

##### Remarks.

In the description of *Xylographus lucasi*, the authors did not have access to type specimens of *Xylographus brasiliensis* and stated that its description was vague (Lopes-Andrade and Lawrence 2003). After we examined the available type of *Xylographus brasiliensis*, a female located in the MNHN, we observed there is no difference between it and female paratypes of *Xylographus lucasi*. We have located in the MNHN a male specimen collected in “Goyaz” (which may correspond to the current state of Goiás or to Tocantins), a historical specimen but not from the original type series of *Xylographus brasiliensis*. We dissected it and compared the sclerites of abdominal terminalia to those of male paratypes of *Xylographus lucasi*, and they are exactly the same. The species is widespread in the tropical South America and the type localities of both names are within its known range (pers. obs.).

#### Xylographus bynoei

Blair, 1940

http://species-id.net/wiki/Xylographus_bynoei

Xylographus bynoei Blair 1940: 131. Type-locality: northwest coast of Australia.

##### Type series.

AUSTRALIA: male holoype (NHM), labeled: “[faded blue disc] N. Holl. [above] 44.4 [below] [handwritten] \ [red disc] Holotype [printed] \ *Xylographus bynoei* Blair Type det. K.G. Blair 1939 [handwritten]”; 2 male and 2 female paratypes (NHM), labeled: “[yellow disc] Paratype [printed] \ N.W. Australia pres. By B. Bynoe, R.N. Surgeon on H.M.S. Beagle. See Stokes, Voyage of Discoveries. 1846 [handwritten]”; 4 female paralectotypes (NHM), labeled: “Australia 44.4 [handwritten] \ [yellow disc] Paratype [printed]”.

#### Xylographus ceylonicus

Ancey, 1876

http://species-id.net/wiki/Xylographus_ceylonicus

Xylographus ceylonicus Ancey 1876: 85. Type-locality: Point de Galle, Ceylon (Sri Lanka).

##### Type series.

SRI LANKA: male lectotype (MNHN), here designated, labeled: “*Xylographus ceylonicus* Ancey, n. sp. Ceylan (Pointe de Galle) Types [handwritten] \ [red label] LECTOTYPE *Xylographus Ceylonicus* Ancey [handwritten]”; 6 males, 1 female and 7 specimens of undetermined gender, all paralectotypes (MNHN), labeled: “*Xylographus Ceylonicus* Ancey, n. sp. Ceylan (Pointe de Galle) Types [handwritten] \ [yellow label] PARALECTOTYPE *Xylographus ceylonicus* Ancey [handwritten]”; 3 male and 1 female paralectotypes (MNHN), labeled: “CEYLAN [printed] \ type [handwritten] \ Syntypes [handwritten] \ Ceylan Pointe de Galles [handwritten] \ *Xylographus ceylonicus* Ancey [handwritten] \ [yellow label] PARALECTOTYPE *Xylographus ceylonicus* Ancey [handwritten]”; 7 paralectotypes of undetermined gender (MNHN), labeled: “*Xylographus Ceylonicus* Ancey Ceylan [handwritten] \ Ex. Coll. REITTER [printed] \ [yellow label] PARALECTOTYPE *Xylographus ceylonicus* Ancey [handwritten]”; 2 paralectotypes of undetermined gender (MFNB), labeled: “*Xylographus Ceylonicus* Ancey Ceylon Ancey Type [handwritten] \ Coll. L.W. Schaufuss [printed] \ [red label] ? SYNTYPUS *Xylographus ceylonicus* Ancey 1876 labelled by MNHUB 1998 [printed] \ [yellow label] PARALECTOTYPE *Xylographus ceylonicus* Ancey [handwritten]”; 2 paralectotypes of undetermined gender (MFNB), labeled: “*Xylographus Ceylonicus* Ancey Ceylan [handwritten] \ [red label] ? SYNTYPUS *Xylographus ceylonicus* Ancey 1876 labelled by MNHUB 1998 [printed] \ [yellow label] PARALECTOTYPE *Xylographus ceylonicus* Ancey [handwritten]”; 2 paralectotypes of undetermined gender (MFNB), labeled: “*Xylographus Ceylonicus* Ancey Ceylan [handwritten] \ ex Coll. Hiller [handwritten] \ [red label] ? SYNTYPUS *Xylographus ceylonicus* Ancey 1876 labelled by MNHUB 1998 [printed] \ [yellow label] PARALECTOTYPE *Xylographus ceylonicus* Ancey [handwritten]”.

#### Xylographus contractus

Mellié, 1849

http://species-id.net/wiki/Xylographus_contractus

Xylographus contractus Mellié 1849: 227, pl. 9, fig. 20. Type-locality: Brazil.

##### Type series.

BRASIL: female lectotype (MNHN), here designated, labeled: “[green disc] *Xylographus Contractus* Bresil Lap. Cast. 72 [handwritten] \ [red label] LECTOTYPE *Xylographus contractus* Mellié [handwritten]”; 1 female paralectotype (MNHN), labeled: “[green disc] *Xylographus contractus* Bresil [unreadable] 83 [handwritten] \ [yellow label] PARALECTOTYPE *Xylographus contractus* Mellié [handwritten]”.

#### Xylographus corpulentus

Mellié, 1849

http://species-id.net/wiki/Xylographus_corpulentus

Xylographus corpulentus Mellié 1849: 225, pl. 9, fig. 19. Type-locality: Peru.
Xylographus lemoulti Pic 1916: 4. **syn. n.** Type-locality: St-Laurent du Maroni, French Guiana.
Xylographus richardi Mellié 1849: 226. **syn. n.** Type-locality: Cayenne, French Guiana.

##### Type series.

PERU: male lectotype (MNHN), here designated, labeled: “[green label] ♂ [handwritten] \ Mellié vidit [handwritten] \ [red label] LECTOTYPE *Xylographus corpulentus* Mellié [handwritten]”; 1 female paralectotype (MNHN), labeled: “[green label] ♀ [handwritten] \ Mellié vidit [handwritten] \ [yellow label] PARALECTOTYPE *Xylographus corpulentus* Mellié [handwritten]”; 1 female paralectotype (MNHM), labeled: “[green label] ♀ [handwritten] \ Mellié vidit [handwritten] \ *Corpulentus* Mell. (Coll. Chevrolat) [handwritten] \ [yellow label] PARALECTOTYPE *Xylographus corpulentus* Mellié [handwritten]”; 1 male and 2 female paralectotypes (MNHN), labeled: “[green disc] *Xylographus Corpulentus* Perou Lap. Cast. 72 [handwritten] \ [yellow label] PARALECTOTYPE *Xylographus corpulentus* Mellié [handwritten]”; 1 male paralectotype (MNHN), labeled: “[green disc] *Xylographus Corpulentus* Kunze Perou T. Cast. 83 [handwritten] \ [yellow label] PARALECTOTYPE *Xylographus corpulentus* Mellié [handwritten]”; 1 male and 2 female paralectotypes (MNHN), labeled: “[green label] Kunze 19 [handwritten] \ [green label] *Cis Corpulentus* Kunze Perou [handwritten] \ [yellow label] PARALECTOTYPE *Xylographus corpulentus* Mellié [handwritten]”.

##### Type material of junior synonyms.

FRENCH GUIANA: male lectotype (MNHN) of *Xylographus lemoulti* Pic, 1916, here designated, labeled: “NOVEMBRE [printed] \ [green label] GUYANE FRANÇAISE St-LAURENT du MARONI [printed] \ [green label] COLL LE MOULT [printed] \ Type [handwritten] \ [red label] LECTOTYPE *Xylographus lemoulti* Pic [handwritten]”; 3 male and 2 female paralectotypes (MNHN), labeled: “NOVEMBRE [printed] \ [green label] GUYANE FRANÇAISE St-LAURENT du MARONI [printed] \ [green label] COLL LE MOULT [printed] \ [yellow label] PARALECTOTYPE *Xylographus lemoulti* Pic [handwritten]”; 2 male and 1 female paralectotypes (MNHN), labeled: “NOVEMBRE [printed] \ GUYANE FRANÇAISE St-LAURENT du MARONI [printed] \ COLL LE MOULT [printed] \ [yellow label] PARALECTOTYPE *Xylographus lemoulti* Pic [handwritten]”; 1 male paralectotype (MNHN), labeled: “NOVEMBRE [printed] \ GUYANE FRANÇAISE St-LAURENT du MARONI [printed] \ COLL LE MOULT [printed] \ [yellow label] PARALECTOTYPE *Xylographus lemoulti* Pic [handwritten]”; 3 male and 4 female paralectotypes (MNHN), labeled: “OCTOBRE [printed] \ GUYANE FRANÇAISE St-LAURENT du MARONI [printed] \ COLL LE MOULT [printed] \ [yellow label] PARALECTOTYPE *Xylographus lemoulti* Pic [handwritten]”; 1 male and 3 female paralectotypes (MNHN), labeled: “JUIN [printed] \ [green label] GUYANE FRANÇAISE St-LAURENT du MARONI [printed] \ [green label] COLL LE MOULT [printed] \ [yellow label] PARALECTOTYPE *Xylographus lemoulti* Pic [handwritten]”; 1 male paralectotype (MNHN), labeled: “MAI [printed] \ GUYANE FRANÇAISE St-LAURENT du MARONI [printed] \ COLL LE MOULT [printed] \ [yellow label] PARALECTOTYPE *Xylographus lemoulti* Pic [handwritten]”.

##### Remarks.

There are several type specimens of species described by Mellié deposited in historical collections of the MHNG and the MNHN. In the MHNG, these types are in the A. Melly collection, who has a surname similar to that of J. Mellié but shall not be confounded. We did not find type material of *Xylographus richardi* in the Chevrolat collection of MNHN. We located a female specimen from Colombia in the Melly collection of MHNG named as *Xylographus richardi*. Mellié (1849) mentioned he has examined specimens from both the Chevrolat and the Melly collections, therefore there is a possibility that this single specimen we located in the Melly collection is a syntype, but we cannot assure this. We compared this female with female paralectotypes of *Xylographus corpulentus* and *Xylographus lemoulti* and they are exactly the same. Mellié (1849) provided few differences between *Xylographus corpulentus* and *Xylographus richardi*, stating that they resemble each other “pour la taille et la forme”, with *Xylographus richardi* being more punctate. We believe the description of *Xylographus richardi* was based on a female specimen, because the pronotal surface between punctures is described as being finely rugose. We have observed that it is common in female *Xylographus* species to have pronotal surface distinctly more rugose than that of males. The type of *Xylographus corpulentus* was described as being black, while the one of *Xylographus richardi* was described as reddish. It is a common variation found in *Xylographus corpulentus*, in which teneral adults may be reddish (pers. obs.). Pic (1916b) mentioned that *Xylographus lemoulti* differs from *Xylographus richardi* in the coloration and pronotal shape, again a consequence of the fact that the description of *Xylographus richardi* was based in a teneral adult female. The type-localities of *Xylographus lemoulti* and *Xylographus richardi* are approximately 200 Km apart and both are in the coast of French Guiana.

#### Xylographus gibbus

Mellié, 1849

http://species-id.net/wiki/Xylographus_gibbus

Xylographus gibbus Mellié 1849: 228. Type-locality: Colombia.

##### Type series.

COLOMBIA: female lectotype (MNHN), here designated, labeled: “[green disc] *Xylographus gibbus* Klg. Mell. Colomb. T. Cast. 72 [handwritten] \ [green label] *Cis gibbus* Klug [handwritten] \ *Xylographus gibbus* Reiche \ MUSEUM PARIS COLL. DE MARSEUL 2842-00 [printed] \ [red label] LECTOTYPE *Xylographus gibbus* Mellié [handwritten]”; 1 male paralectotype (MHNG), labeled: “*Gibbus* Klug Colombie Klug Mellié [handwritten] \ [yellow label] PARALECTOTYPE *Xylographus gibbus* Mellié [handwritten]”.

#### Xylographus globipennis

Reitter, 1911

Xylographus globipennis Reitter 1911: 52. Type-locality: Gorbatuco, Eritrea.

##### Type series.

See Sandoval-Gómez et al. (2011).

#### Xylographus hypocritus

Mellié, 1849

http://species-id.net/wiki/Xylographus_hypocritus

Xylographus hypocritus Mellié 1849: 221, pl. 9, fig. 16. Type-locality: Madagascar.

##### Type series.

MADAGASCAR: male lectotype (MNHN), here designated, labeled: “*Hypocritus* Dup. Madagascar. [handwritten] \ Ex-Musæo Mniszech [printed] \ [red label] LECTOTYPE *Xylographus hypocritus* Mellié [handwritten]”; 1 female paralectotype (MNHN), labeled: “Madagascar [handwritten] \ Ex-Musæo Mniszech [printed] \ [yellow label] PARALECTOTYPE *Xylographus hypocritus* Mellié [handwritten]”.

#### Xylographus madagascariensis

Mellié, 1849

http://species-id.net/wiki/Xylographus_madagascariensis

Xylographus madagascariensis Mellié 1849: 224, pl. 9, fig. 18. Type-locality: Madagascar.
Xylographus eichelbaumi Reitter 1908: 119. s**syn. n.** Type-locality: Amani, Tanzania.
Xylographus rufipennis Pic 1934: 14. **syn. n.** Type-locality: Gura, Kenya.
Xylographus seychellensis Scott 1926: 10. **syn. n.** Type-locality: Mahe, Seychelles.
Xylographus tarsalis Fåhraeus 1871: 670. **syn. n.** Type-locality: Caffraria (Eastern Cape of South Africa).

##### Type series.

MADAGASCAR: male lectotype (MNHN), here designated, labeled: “*Madagascariensis* Dup. Madagascar.[handwritten] \ Ex-Musæo Mniszech [printed] \ [red label] LECTOTYPE *Xylographus madagascariensis* Mellié [handwritten]”.

##### Type material of junior synonyms.

KENYA: female lectotype (NHM) of *Xylographus rufipennis* Pic, 1934, here designated, labeled: “[red disc]Type [printed] \ R. E, DENT GURA R, 7500 AUG 1929 [printed] \ *Xylographus rufipennis* n. sp. [handwritten] \ Pres. By Imp. Inst. Ent B. M. 1934-42. [printed] \ [red label] LECTOTYPE *Xylographus rufipennis* Pic [handwritten]”; 1 female paralectotype (MNHN), labeled: “R. E, DENT GURA R, 7500 AUG 1929 [printed] \ *Xylographus rufipennis* n. sp [handwritten] \ ex. British museum [handwritten] \ [yellow label] PARALECTOTYPE *Xylographus rufipennis* Pic [handwritten]”. SEYCHELLES: male lectotype (NHM) of *Xylographus seychellensis* Scott 1926, here designated, labeled: “[purple disc] LECTOTYPE [printed] \ Mahe, 1908-9 Seychelles Exp. [printed] \ Percy Sladen Trust Exped. Brit. Mus. 1926-246. [printed] \ *Xylographus seychellensis*, Scott TYPE. ♂. [handwritten] \ Figured specimen [printed] (outline whole vis) [handwritten] \ TYPE [printed] \ [red label] LECTOTYPE *Xylographus seychellensis* Scott [handwritten]”. SOUTH AFRICA: male lectotype (NHRS) of *Xylographus tarsalis* Fåhraeus 1871, labeled: “Caffraria [printed] \ J. Wahlb. [printed] \ ♂ [printed] \ [red label] Lectotype ♂ *Xylographus tarsalis* FÅHR. Det. Julio Ferrer 1995 [handwritten] \ [green label] Riksmuseum Stockholm [printed]”; 1 male and 1 female paralectotypes (NHRS), labeled: “Caffraria [printed] \ J. Wahlb. [printed] \ [red label] Paralectotype *Xylographus tarsalis* FÅHR. Det. Julio Ferrer 1995 [handwritten] \ [green label] Riksmuseum Stockholm [printed]”. TANZANIA: female holotype (NHMW) of *Xylographus eichelbaumi* Reitter 1908, labeled: “6. [handwritten] \ Amani [printed] \ D. O. Afrika Eichelbaum’03 [printed] \ 13 April 1903 in *Fomes nigrolachatus* [handwritten] \ Amani. Deutsch Ostafr. [handwritten] \ *Xylographus eichelbaumi* m. Typ. 1907. [handwritten] \ Eichelbaumi Usambara Reitt. [handwritten] \ [red label] HOLOTYPE *Xylographus eichelbaumi* Reitt. [handwritten]”.

##### Remarks.

Scott (1926) stated he has not examined the type of *Xylographus madagascariensis* and that he described *Xylographus seychellensis* with some hesitation. If he had examined the known male type of *Xylographus madagascariensis*, he would have observed that it was just slightly more elongate than the specimens he had at hand, with no other differences. Such a small difference in body elongation is expected to occur in *Xylographus* species with broad geopraphical distribution (see, for instance, the known variation in *Xylographus globipennis*; Sandoval-Gómez et al. 2011). In order to make sure they were all conspecifics, we dissected named male *Xylographus seychellensis* compared to the type and also the lectotype of *Xylographus madagascariensis*, and we observed the sclerites of abdominal terminalia to be exactly the same. The lectotype of *Xylographus tarsalis* is a male *Xylographus madagascariensis* with weak secondary sexual characteristics. Ferrer (1997) stated that two female paralectotypes of *Xylographus tarsalis* were deposited in NHRS. After studying the material, we have seen that they are a male and a female instead. The names *Xylographus eichelbaumi* and *Xylographus rufipennis* were based on females, which clearly correspond to females named *Xylographus madagascariensis* that we examined.

#### Xylographus nitidissimus

Pic, 1916

http://species-id.net/wiki/Xylographus_nitidissimus

Xylographus nitidissimus Pic 1916: 13. Type-locality: São Tomé, São Tomé and Príncipe.
Xylographus longicollis Pic 1922: 8. **syn. n.** Type-locality: Dahomey, Benin.

##### Type series.

SÃO TOMÉ AND PRÍNCIPE: male lectotype (MNHN) of *Xylographus nitidissimus* Pic 1916, here designated, labeled: “[green label] San. Thomé [printed] \ n. sp. [handwritten] \ type [handwritten] \ *nitidissimus* Pic [handwritten] \ [red label] LECTOTYPE *Xylographus nitidissimus* Pic [handwritten]”; 1 male paralectotype (MNHN), labeled: “type [handwritten] \ [yellow label] PARALECTOTYPE *Xylographus nitidissimus* Pic [handwritten]”; 4 male and 4 female paralectotypes (MNHN), labeled: “San Thomé [handwritten] \ [yellow label] PARALECTOTYPE *Xylographus nitidissimus* Pic [handwritten]”.

##### Type material of the junior synonym.

BENIN: female lectotype (MNHN) of *Xylographus longicollis* Pic, 1922, here designated, labeled: “? Dahomey [handwritten] \ Provenance ? [handwritten] \ *longicollis* n. sp. [handwritten] \ [red label] LECTOTYPE *Xylographus longicollis* Pic [handwritten]”.

##### Remarks.

We observed that the female lectotype of *Xylographus longicollis* is a female of *Xylographus nitidissimus*.

#### Xylographus perforatus

Gerstaecker, 1871

http://species-id.net/wiki/Xylographus_perforatus

Xylographus perforatus Gerstaecker 1871: 57. Type-locality: Tanzania, Zanzibar.

##### Type series.

TANZANIA: male lectotype (MFNB), here designated, labeled: “[green label] *perforatus* Gerst.* Sansibar Cooke [handwritten] \ 56743 [printed] \ [blue label] Hist.-Coll. (Coleoptera) Nr. 56743 (1. Ex) *Xylographus perforatus* Gerst. Sansibar, Cooke Zool. Mus. Berlin [printed] \ [red label] SYNTYPUS *Xylographus perforatus* Gerstaecker 1871 labelled by MNHUB 1998 \ [red label] LECTOTYPE *Xylographus perforatus* Gerstaecker [handwritten]”; 2 male and 3 female paralectotypes (MFNB), labeled: “[blue label] Hist.-Coll. (Coleoptera) Nr. 56743 (1.-5. Ex) *Xylographus perforatus* Gerst. Sansibar, Cooke Zool. Mus. Berlin [printed] \ [red label] SYNTYPUS *Xylographus perforatus* Gerstaecker 1871 labelled by MNHUB 1998 \ [yellow label] PARALECTOTYPE *Xylographus perforatus* Gerstaecker [handwritten]”; 1 female paralectotype (MNHN), labeled: “Zanzibar, C. Cooke. [printed] \ [blue label] MUSEUM PARIS Collection Léon Fairmaire 1906 [printed] \ *Xylographus perforatus* Gerst. [handwritten] P. Lesne vid. [printed] \ [yellow label] PARALECTOTYPE *Xylographus perforatus* Gerstaecker [handwritten]”; 1 female paralectotype (MNHN), labeled: “Zanzibar, C. Cooke. [printed] \ Bragance (Para) M. de Mathan [printed] \ Kein Scolytide [handwritten] \ [yellow label] PARALECTOTYPE *Xylographus perforatus* Gerstaecker [handwritten]”; 1 male and 1 female paralectotype (MNHN), labeled: “Zanzibar, C. Cooke. [printed] \ *Xylographus perforatus* Gerst. Zanzib. [handwritten] \ [yellow label] PARALECTOTYPE *Xylographus perforatus* Gerstaecker [handwritten]”; 1 male and 1 female paralectotypes (MCZ), labeled: “Zanzibar, C. Cooke. [printed] \ [black disc] \ *Xylographus perforatus* 140 Gerstaecker [printed] \ [yellow label] PARALECTOTYPE *Xylographus perforatus* Gerstaecker [handwritten]”.

#### Xylographus porcus

Gorham, 1886

http://species-id.net/wiki/Xylographus_porcus

Xylographus porcus Gorham 1886: 355. Type-locality: Teleman, Guatemala.

##### Type series.

GUATEMALA: male lectotype (NHM), here designated, labeled: “[purple disc] LECTOTYPE [printed] \ Teleman, Vera Paz. Champion. [printed] \ Type. [printed] \ *Cis*, *porcus* [handwritten] \ B.C.A., Col., III (2). *Xylographus porcus*. [printed] \ [red label] LECTOTYPE *Xylographus porcus* Gorh. [handwritten]”; 1 male and 2 female paralectotypes (MNHN), labeled: “Teleman, Vera Paz. Champion. [printed] \ *Xylographus porcus*, Gorh [handwritten] \ [yellow label] PARALECTOTYPE *Xylographus porcus* Gorh. [handwritten]”; 2 female paralectotypes (MNHN), labeled: “Zapote, Guatemala. G. C. Champion. [printed] \ [yellow label] PARALECTOTYPE *Xylographus porcus* Gorh. [handwritten]”; 1 male and 2 female paralectotypes (MNHN), labeled: “Pantaleon, 700 ft. Champion. [printed] \ [yellow label] PARALECTOTYPE *Xylographus porcus* Gorh. [handwritten]”.

#### Xylographus punctatus

Mellié, 1849

http://species-id.net/wiki/Xylographus_punctatus

Xylographus punctatus Mellié 1849: 230, pl. 9, fig. 21. Type-locality: Colombia.

##### Type series.

COLOMBIA: male lectotype (MNHN), here designated, labeled: “[green label] ♂ [handwritten] \ Nouv. Grenada [handwritten] \ Coll. Chevrolat [handwritten] \ [red label] LECTOTYPE *Xylographus punctatus* Mellie [handwritten]”; 1 male and 3 female paralectotypes (MNHN), labeled: “Nouv. Grenada [handwritten] \ Coll. Chevrolat [handwritten] \ [yellow label] PARALECTOTYPE *Xylographus punctatus* Mellie [handwritten]”; 1female paralectotype (MNHN), labeled: “Carthagene [handwritten] \ Collect. Chevrolat [handwritten] \ [yellow label] PARALECTOTYPE *Xylographus punctatus* Mellie [handwritten]”; 1 male paralectotype (MNHN), labeled: “[green disc] *Xylographus punctatus* Mell. Colomb. T. Cast. 72 [handwritten] \ [pink disc] punctatus [handwritten] \ punctatus Chev. Colombia [handwritten] \ MUSEUM PARIS COLL DE MARSEUL [printed] \ [yellow label] PARALECTOTYPE *Xylographus punctatus* Mellie [handwritten]”; 1 male paralectotype (MNHN), labeled: “[green disc] *Xylographus punctatus* Colomb. Lap. Cast. 72 [handwritten] \ [green label] Chev^t^. Colomb [handwritten] \ [yellow label] PARALECTOTYPE *Xylographus punctatus* Mellie [handwritten]”.

#### Xylographus ritsemai

Pic, 1921

http://species-id.net/wiki/Xylographus_ritsemai

Xylographus ritsemai Pic 1921: 7. Type-locality: Ceylon (Sri Lanka).

##### Type series.

SRI LANKA: male lectotype (MNHN), here designated, labeled: “*Ritsemai* Pic [handwritten] \ type [handwritten] \ Ceylon. Ancey. [handwritten] \ [red label] LECTOTYPE *Xylographus ritsemai* Pic [handwritten]”; 1 male paralectotype (MNHN), labeled: “*Ritsemai* Pic [handwritten] \ type [handwritten] \ [yellow label] PARALECTOTYPE *Xylographus ritsemai* Pic [handwritten]”.

#### Xylographus rufipes

Pic, 1930

http://species-id.net/wiki/Xylographus_rufipes

Xylographus rufipes Pic 1930: 175. Type-locality: Tucumán, Argentina.

##### Type series.

ARGENTINA: male lectotype (MNHN), here designated, labeled: “Argentina Tucumán 1 Oct. 1929 [printed] \ H. E. Box leg. [printed] \ 2705 [printed] \ *Xylographus rufipes* n. sp. [handwritten] \ [red label] LECTOTYPE *Xylographus rufipes* Pic [handwritten]”; 1 male paralectotype (NHM), labeled: “Argentina Tucumán 1 Oct. 1929 [printed] \ H. E. Box leg. [printed] \ 2709 [printed] \ *Xylographus rufipes*, Pic sp. nov. (det. Pic, per C. Bruch) [handwritten] \ Brit. Mus. 1948-460. [printed] \ [yellow label] PARALECTOTYPE *Xylographus rufipes* Pic [handwritten]”.

#### Xylographus scheerpeltzi

Nobuchi & Wada, 1956

http://species-id.net/wiki/Xylographus_scheerpeltzi

Xylographus scheerpeltzi Nobuchi and Wada 1956: 53, figs. 1–2. Type-locality: Japan.

##### Remarks.

Nobuchi and Wada (1956) described this species based on a series of 28 syntypes from five different localities in Japan. We did not have access to material of Mr. T. Nakane’s collection from the Hokkaido University Museum, so a lectotype for this species is not designated here.

#### Xylographus subopacus

Pic, 1929

http://species-id.net/wiki/Xylographus_subopacus

Xylographus subopacus Pic 1929: 264. Type-locality: Democratic Republic of the Congo, Elisabethville.

##### Type series.

DEMOCRATIC REPUBLIC OF THE CONGO: female lectotype (NHM), here designated, labeled: “[purple disc] LECTOTYPE [printed] \ BELGIAN CONGO. 18 m. S. W. of Elizabethville. 24.iii.1928. Dr. H. S. Evans. [printed] \ Pres. by Imp. Inst. Ent. Brit. Mus. 1932-147. [printed] \ [red label] LECTOTYPE *Xylographus subopacus* Pic [handwritten]”; 1 female paralectotype (MNHN), labeled: “BELGIAN CONGO. 18 m. S. W. of Elizabethville. 1928. Dr. H. S. Evans. [printed] \ *Xylographus subopacus* n. sp. [handwritten] \ [yellow label] PARALECTOTYPE *Xylographus subopacus* Pic [handwritten]”.

#### Xylographus subsinuatus

Pic, 1916

http://species-id.net/wiki/Xylographus_subsinuatus

Xylographus subsinuatus Pic 1916: 4. Type-locality: Madagascar.
Xylographus rufescens Pic 1921: 7. **syn. n.** Type-locality: Bourbon Island (Reunion).

##### Type series.

MADAGASCAR: male lectotype (MNHN), here designated, labeled: “MADAGASCAR Plantations du Sambirano COLLECTION LE MOULT [printed] \ [red label] Coll. C [handwritten] \ Type [handwritten] \ *subsinuatus* Pic [handwritten] \ [red label] LECTOTYPE *Xylographus subsinuatus* Pic [handwritten]”; 6 males, 3 females, 17 specimens of undetermined gender, all paralectotypes (MNHN), labeled: “MADAGASCAR Plantations du Sambirano COLLECTION LE MOULT [printed] \ [red label] Coll. C [handwritten] \ [yellow label] PARALECTOTYPE *Xylographus subsinuatus* Pic [handwritten]”.

##### Type material of the junior synonym.

REUNION: male lectotype (MNHN) of *Xylographus rufescens* Pic 1921, here designated, labeled: “Ile Bourbon n. sp. [handwritten] \ type [handwritten] \ *rufescens* Pic [handwritten] \ [red label] LECTOTYPE *Xylographus rufescens* Pic [handwritten]”.

##### Remarks.

We observed that the lectotype of *Xylographus rufescens* is a small teneral male of *Xylographus subsinuatus*. We dissected the types and compared the sclerites of abdominal terminalia, which are identical.

#### Xylographus suillus

Gorham, 1886

http://species-id.net/wiki/Xylographus_suillus

Xylographus suillus Gorham 1886: 355, pl. 13, figs. 21, 21a. Type-locality: Teleman, Guatemala.

##### Type series.

GUATEMALA: male lectotype (NHM), here designated, labeled: “[purple disc] LECTOTYPE [printed] \ Type. Sp. figured [printed] \ Teleman, Vera Paz. Champion. [printed] \ *Xylographus suillus*, Gorh. [handwritten] \ B.C.A., Coll., III (2). *Xylographus suillus*, Gorh. [printed] \ [red label] LECTOTYPE *Xylographus suillus* Gorh. [handwritten]”; 2 male and 4 female paralectotypes (MNHN), labeled: “Teleman, Vera Paz. Champion. [printed] \ [pink label] In boleti attached to manaca palm [handwritten] \ *Xylographus suillus*, Gor. [handwritten] \ [yellow label] PARALECTOTYPE *Xyloraphus suillus* Gorh. [handwritten]”.

#### Xylographus tomicoides

Reitter, 1902

http://species-id.net/wiki/Xylographus_tomicoides

Xylographus tomicoides Reitter 1902: 47. Type-locality: Chabarowka, Amur, Russia.

##### Type series.

RUSSIA: male lectotype (MNHN), here designated, labeled: “Amur [handwritten] \ *tomicoides* m. 1902 [handwritten] \ [red label] LECTOTYPE *Xylographus tomicoides* Reitter [handwritten]”.

#### Excluded species

##### Cis renominatus nom. n.

Xylographus dentatus Pic 1922: 7. Secondary junior homonym of *Cis dentatus* Mellié, 1849. Type-locality: Republic of the Congo.

###### Type series.

REPUBLIC OF THE CONGO: male lectotype (MNHN), here designated, labeled: “Franz. Congo [printed] \ *dentatus* n. sp. [handwritten] \ *Cis* sp. A. Kompantsev det. 2010 [handwritten] \ [red label] LECTOTYPE *Xylographus dentatus* Pic [handwritten] \ [green label] *Cis renominatus* Sandoval-Gómez, Lópes-Andrade & Lawrence nom. n.”; 6 male and 2 female paralectotypes (MNHN), labeled: “Franz. Congo [printed] \ [yellow label] PARALECTOTYPE *Xylographus dentatus* Pic [handwritten] \ [green label] *Cis renominatus* Sandoval-Gómez, Lópes-Andrade & Lawrence nom. n.”.

###### Remarks.

*Xylographus dentatus* Pic, 1922 is transferred to the genus *Cis*, but *Cis dentatus* (Pic, 1922) becomes a junior secondary homonym of *Cis dentatus* Mellié, 1849. The replacement name proposed here means “renamed”.

##### Paratrichapus fultoni

(Broun, 1886) comb. n.

http://species-id.net/wiki/Paratrichapus_fultoni

Cis fultoni Broun 1886: 904. Type-locality: West Taieri, New Zealand.
Xylographus fultoni (Broun, 1886) Kuschel 1990: 62.

###### Type series.

NEW ZEALAND: male lectotype (NHM), here designated, labeled: “1614 [handwritten] \ Taieri [printed] \ New Zealand. Broun Coll. Brit. Mus. 1922-482 [printed] \ *Cis fultoni* [handwritten] \ *Rhopalodontus fultoni* Broun. K. Paviour-Smith det. 1966 [handwritten] \ [red label] LECTOTYPE *Cis fultoni* Broun [handwritten]”; 1 female paralectotype (NHM), labeled: “1614 [handwritten] \ Taieri [printed] \ New Zealand. Broun Coll. Brit. Mus. 1922-482 [printed] \ *Cis fultoni* [handwritten] \ *Rhopalodontus fultoni* Broun. K. Paviour-Smith det. 1966 [handwritten] \ [yellow label] PARALECTOTYPE *Cis fultoni* Broun [handwritten]”.

##### Paratrichapus javanus

(Pic, 1937) comb. n.

http://species-id.net/wiki/Paratrichapus_javanus

Xylographus javanus Pic 1937: 304. Type-locality: Goenoeng Tangkoeban Prahoe, Java, Indonesia.
Xylographus javanus var. *rufomarginatus*Pic 1937: 304. Junior synonym. Type-locality: Goenoeng Tangkoeban Prahoe, Java, Indonesia.

###### Type series.

INDONESIA: male lectotype (MNHN), here designated, labeled: “F. C. DRESCHER G. Tangkoeban Prahoe 4000.5000 Voet. Preanger. Java 31.x.1934 [printed] \ ex *Fomes melanopurus* Mont. [printed] \ n. sp. diffère de *Xylographus ceylonicus* Ancey par la forme plus allongée, le thorax moins court, plus fortement rétréci en avant, les élytres sans pli huméral brillant [handwritten] \ [red label] LECTOTYPE *Xylographus javanus* Pic [printed] \ *Paratrichapus javanus* (Pic, 1937) comb. n. Sandoval-Gómez, Lopes-Andrade & Lawrence [handwritten]”; 1 male paralectotype (MNHN), labeled: “F. C. DRESCHER G. Tangkoeban Prahoe 4000.5000 Voet. Preanger. Java 31.x.1934 [printed] \ ex *Fomes melanopurus* Mont. [printed] \ [yellow label] PARALECTOTYPE *Xylographus javanus* Pic [printed] \ *Paratrichapus javanus* (Pic, 1937) comb. n. Sandoval-Gómez, Lopes-Andrade & Lawrence [handwritten]”; 1female paralectotype (MNHN), labeled: “F. C. DRESCHER G. Tangkoeban Prahoe 4000.5000 Voet. Preanger. Java 22.i.1935 [printed] \ ex *Fomes melanopurus* Mont. [printed] \ *Xylographus javanus* n. sp. [handwritten] \ [yellow label] PARALECTOTYPE *Xylographus javanus* Pic [printed] \ *Paratrichapus javanus* (Pic, 1937) comb. n. Sandoval-Gómez, Lopes-Andrade & Lawrence [handwritten]”.

## Supplementary Material

XML Treatment for Xylographus anthracinus

XML Treatment for Xylographus bicolor

XML Treatment for Xylographus bostrichoides

XML Treatment for Xylographus brasiliensis

XML Treatment for Xylographus bynoei

XML Treatment for Xylographus ceylonicus

XML Treatment for Xylographus contractus

XML Treatment for Xylographus corpulentus

XML Treatment for Xylographus gibbus

XML Treatment for Xylographus globipennis

XML Treatment for Xylographus hypocritus

XML Treatment for Xylographus madagascariensis

XML Treatment for Xylographus nitidissimus

XML Treatment for Xylographus perforatus

XML Treatment for Xylographus porcus

XML Treatment for Xylographus punctatus

XML Treatment for Xylographus ritsemai

XML Treatment for Xylographus rufipes

XML Treatment for Xylographus scheerpeltzi

XML Treatment for Xylographus subopacus

XML Treatment for Xylographus subsinuatus

XML Treatment for Xylographus suillus

XML Treatment for Xylographus tomicoides

XML Treatment for Cis renominatus

XML Treatment for Paratrichapus fultoni

XML Treatment for Paratrichapus javanus
